# Late Subcutaneous Infection Caused by *Serratia marcescens* Following Hyaluronic Acid Injection: A Case Report and Systemic Review

**DOI:** 10.1111/jocd.16571

**Published:** 2024-09-17

**Authors:** Yihan Zhang, Yansi Lyu, Tingyin Lin, Luotai Chen, Zhuolin Liu, Yanting Ou, Xiangwen Xu, Mengfan Wu, Lin Luo, Jun Feng, Dandan Liu

**Affiliations:** ^1^ Department of Plastic and Reconstructive Surgery Peking University Shenzhen Hospital Shenzhen Guangdong P.R. China; ^2^ Shantou University Medical College Shantou P.R. China; ^3^ Department of Dermatology Shenzhen University General Hospital Shenzhen Guangdong P.R. China; ^4^ Department of Plastic and Reconstructive Surgery Shenzhen Xinhua Hospital Shenzhen P.R. China

**Keywords:** case report, cutaneous infection, *Serratia marcescens*, *Serratia marcescens* infection, systemic review

## Abstract

**Background:**

The field of cosmetic filler injection has experienced rapid development over the past two decades, especially in facial augmentation utilizing hyaluronic acid (HA) fillers. Gram‐negative bacteria are found to be the main pathogens of infective nodules after HA injection. The occurrence of cutaneous infections attributed to *Serratia marcescens* is exceedingly rare and predominantly noted in patients with compromised immune systems.

**Aims:**

To summarize the clinical features, diagnosis, and treatment of subcutaneous infection caused by *Serratia marcescens* following hyaluronic acid injection.

**Patients/Methods:**

A rare case of cutaneous *Serratia marcescens* infection following hyaluronic acid injection was presented. A comprehensive review of the published literature describing the management of skin infection caused by *S. marcescens* in immunocompetent patients was then conducted, which encompassed three case series and eight case reports published between 1999 and 2017. Data extraction included information on authors, gender, age, signs and symptoms, previous treatment, corresponding management strategies, and follow‐up duration.

**Results:**

*Serratia marcescens* were isolated in abscesses (*n* = 6, 35.29%), painful nodules (*n* = 2, 11.76%), ulcers (*n* = 6, 35.29%), and others (*n* = 3, 17.65%). In cases providing salvage plans (*n* = 11), quinolones were shown to be the most effective antibiotics for salvage, with eight full recoveries (72.73%), and trimethoprim‐sulfamethoxazole was the second most useful antibiotic (18.18%).

**Conclusions:**

With the help of pathogen examination and drug‐sensitive tests, sensitive aminoglycosides, quinolone (especially moxifloxacin), or TMP‐SMX for at least 2 weeks can be considered as the first‐line treatment of late subcutaneous infection caused by *Serratia marcescens* following hyaluronic acid injection.

## 1 Background

The field of cosmetic filler injection has experienced rapid development over the past two decades, especially in facial augmentation utilizing hyaluronic acid (HA) fillers, renowned for their safety and high efficacy. As a rare complication, late tissue nodules usually arise following HA injection. The formation of such nodules has been proven to be relative to the presence of filler, the immune system of the patient, and infection [1, 2]. For infective nodules, gram‐negative bacteria have been found to be the main representative pathogen [3].

*Serratia marcescens* (*S. marcescens*) is a gram‐negative, encapsulated, motile, anaerobic, non‐sporulating bacillus belonging to the Enterobacteriaceae family. This bacterium typically incites nosocomial infections, such as respiratory and genitourinary infections. The occurrence of cutaneous infections attributed to *S. marcescens* is exceedingly rare and predominantly noted in patients with compromised immune systems, chronic renal failure, neoplasms, and those undergoing systemic corticosteroid therapy [4, 5]. Cutaneous infections caused by *S. marcescens* among immunocompetent patients are uncommon.

This paper presents a rare case and reviews the existing literature on skin infection subsequent to cosmetic injection caused by *S. marcescens* in immunocompetent patients.

## 2 Method

**FIGURE 1:**
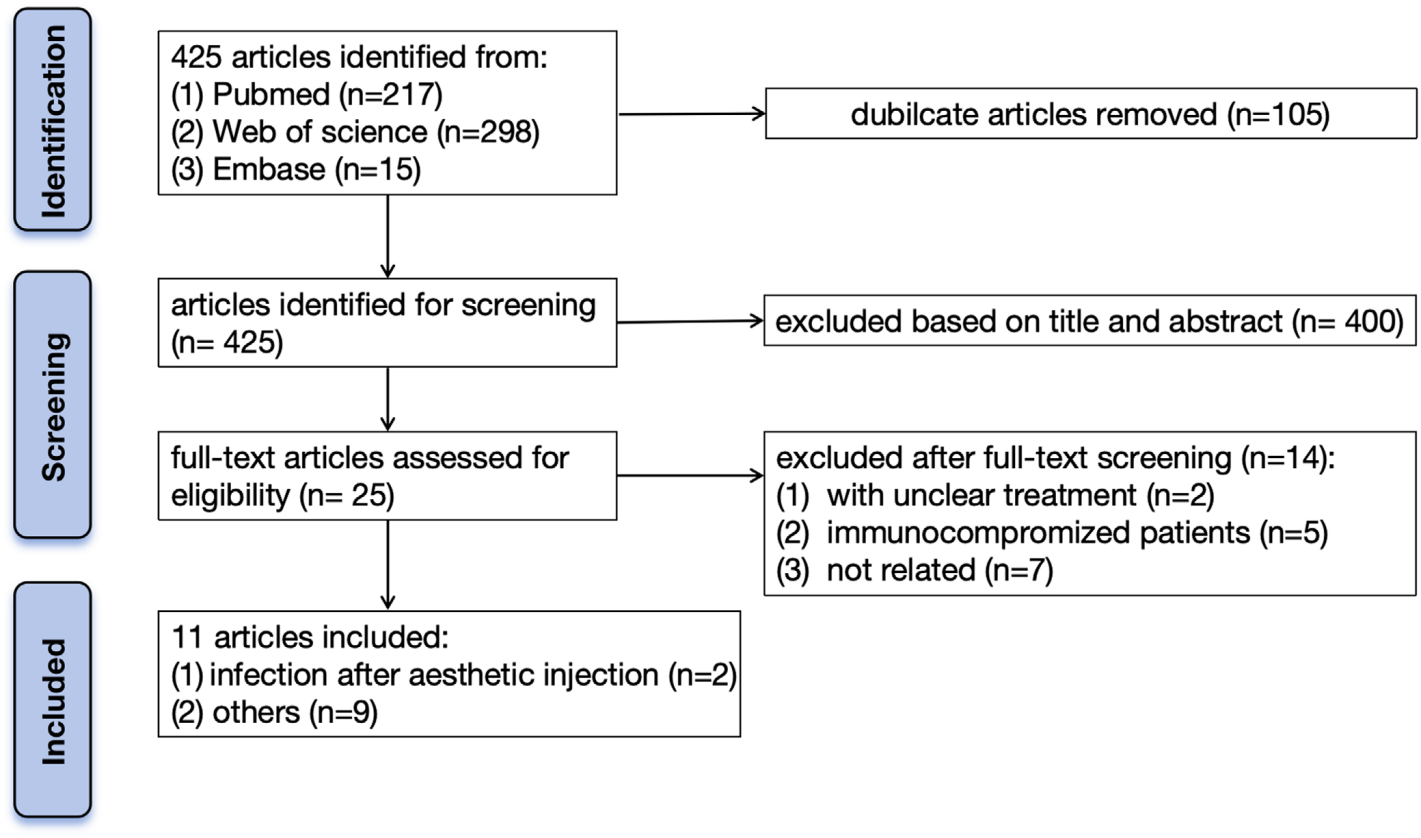
Flow diagram of the study selection process.

A comprehensive review of the published literature describing the management of skin infection caused by *S. marcescens* in immunocompetent patients was conducted. The PubMed, Medline, and Embase databases were systematically searched from their inception to February 10, 2024. The search strategy involved keywords such as “*Serratia marcescens*” and “skin infection.” Following the removal of duplicates, 425 references were screened based on titles and abstracts. Full texts of 25 references were retrieved, and 11 studies were chosen for inclusion in this review based on predefined inclusion and exclusion criteria. Articles reporting the management of skin infection caused by *S. marcescens* in immunocompetent patients were included, while studies that did not address skin infection, focused solely on immunocompromised patients, or lacked clear treatment protocols were excluded. Additionally, references cited in the included articles were also reviewed to identify potentially relevant studies for inclusion. Two authors independently screened all articles according to the established criteria. The screening process is illustrated in Figure 1. This review encompassed three case series and eight case reports published between 1999 and 2017. Data extraction included information on authors, gender, age, signs and symptoms, previous treatment, corresponding management strategies, and follow‐up duration.

## 3 Case Report

**FIGURE 2:**
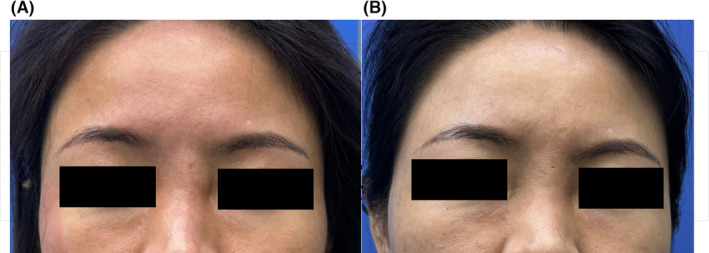
(A) Erythematous inflamed nodule on the glabella area. (B) Clinical findings 1 month after treatment with IV ceftriaxone 1.5 g qd and oral levofloxacin 0.5 g qd for 16 days.

A 44‐year‐old woman presented to the Plastic and Reconstructive Surgery Clinic on December 16, 2023. The patient had a history of erythematous nodules at the root of the nose (Figure 2A), which were on and off for 5 months. Her medical history included thread lift 8 years ago and augmentation rhinoplasty 3 years ago. Two months after augmentation rhinoplasty with polytetrafluoroethylene and ear cartilage autografts, the patient received a hyaluronic acid injection at the roof of the nose to correct nose deviation. The polytetrafluoroethylene and ear cartilage autografts were removed 2 years ago because of an infection. Five months ago, an erythematous nodule appeared tenderly at the site where HA was injected. She then went to a private clinic. Hyaluronic acid (with unclear dosage) was injected into the nodule; the size of the nodule decreased within 1–2 days but relapsed on Day 3. She reported no improvement with the prescribed treatment and believed the eruption was worsening. During the first visit to our clinic, no other accompanying skin lesions, enlarged lymph nodes, or signs of systemic involvement were detected. Late bacterial infection after aesthetic HA augmentation was suspected at that time. Hyaluronic acid (150 U) was then injected into the central area of the nodule. Oral cefuroxime was also administered for 3 days, resulting in the resolution of nodules. But she returned to our clinic 3 weeks later, complaining of a recurrence. Three debridements, each of which was followed by 3 days of oral cefuroxime (0.25 g bid), were administered over the next 50 days, but the inflamed nodule continued to wax and wane. A pathogen examination of the pus was then performed after the incision was drained. It showed infection of *S. marcescens*, which was sensitive to ceftriaxone, ceftazidime, cefepime, cefoperazone sulbactam, ertapenem, amikacin, levofloxacin, tigecycline, and trimethoprim‐sulfamethoxazole (TMP‐SMX). Antimicrobial therapy was administered based on the antibiogram: intravenous ceftriaxone 1.5 g qd and oral levofloxacin 0.5 g qd for 16 days, which resulted in a complete resolution of the lesion. No recurrence was observed 6 months after the completion of therapy (Figure 2B).

## 4 Result of Literature Review

The results of cutaneous infection caused by *S. marcescens* among immunocompetent patients are summarized in Table 1. *Serratia marcescens* were isolated in abscesses (*n* = 6, 35.29%), painful nodules (*n* = 2, 11.76%), ulcers (*n* = 6, 35.29%), and others (*n* = 3, 17.65%). Three cases of *Serratia marcescens*‐related skin infections developed after cosmetic injection, located in the glabellar area, nasolabial fold, and nose, respectively. In cases providing salvage plans (*n* = 11), amoxicillin/clavulanic acid proved to be the least effective antibiotics, with five cases of relapse (45.45%). By contrast, quinolones were shown to be the most effective antibiotics for salvage, with eight full recoveries (72.73%), and trimethoprim‐sulfamethoxazole was the second most useful antibiotic (18.18%).

**TABLE 1:** Cutaneous infection caused by *Serratia marcescens* among immunocompetent patients.

Author	Sex/Age	Risk factor	Site	Signs and symptoms	Previous treatment	Strategy	Follow‐up
Diranzo García J [6]	M/32	Elbow fracture 15 years ago Tattoo 30 days ago	Upper arm	Abscess and fever	Amoxicillin/clavulanic acid 1 g/200 mg + clindamycin 600 mg IV q8h	Debridement and drainage Ciprofloxacin 1.5 g IV + ertapenem 1 g IV qd for 3 days Ciprofloxacin 500 mg PO q12h for 21 days	15 weeks
Rallis E [7]	M/21	N	Chin	Abscess and fever	Amoxicillin/clavulanic acid 625 mg tid for 10 days Topical antiseptics and fusidic acid	Ciprofloxacin 400 mg IV bid for 10 days	5 months
Seo J [8]	F/21	Incision and drainage of cutaneous abscess	Back	Abscess	N	N	N
Seo J [8]	M/42	N	Lower leg	Abscess	N	N	N
Giráldez P [9]	M/40	N	Hand	Abscess	Amoxicillin/clavulanic acid IV Topical mupirocin and fusidic acid	Ciprofloxacin 750 mg PO qd for 15 days Trimethoprim‐sulfamethoxazole 800/160 mg PO q12h for 3 months. Antibiotic treatment was restarted for 2 months because of recurrence 1 month later.	1 year
Park KY [10]	F/62	Artecoll injection 2 years ago; removal of collagen materials along with injection of Juvéderm 3 months ago	Glabellar area and forehead	Abscess	Incisional drainage Amoxicillin‐clavulanate and ceftezole IV Topical mupirocin and fusidic acid	Incisional drainage Trimethoprim‐sulfamethoxazole 800/160 mg PO bid for 3 weeks	1 year
Seo J [8]	M/70	Cosmetic eyelid surgery and filler injection (unclear material)	Nasolabial fold	Painful nodule	N	N	N
Nieves DS [11]	M/45	N	Leg	Painful nodule	IV and topical corticosteroids, penicillin, cephalexin, erythromycin, mupirocin ointment, and pentoxyphylline.	Ofloxacin 400 mg PO bid for 3 weeks. Gentamicin ointment once weekly.	2 years
Sharma V [12]	F/11	Insect bite	Chest	Ulcer subsequent to a burst abscess	Ampicillin + cloxacillin 200 mg IV q6h Metronidazole 50 mg IV q8h Amikacin 120 mg IV qd	Ceftazidime 400 mg IV q8h + amikacin 120 mg IV qd for 10 days Amoxyclav 5 mL bid	2 weeks
Yoshida R [13]	F/54	N	Chin	Ulcer subsequent to an erythematous plaque	Cefdinir and clarithromycin IV Topical gentamicin and clindamycin	Ciprofloxacin 600 mg PO qd for 2 months	18 months
Carlesimo M [14]	M/18	N	Leg	Multiple ulcers subsequent to small pustule	Prednisone 35 mg qd + dapsone 75 mg qd for 6 days a week for 3 months Trimethoprim‐sulfamethoxazole and minocycline after relapsed.	Ciprofloxacin 800 mg IV qd for 5 days Ciprofloxacin 1000 mg PO qd for 30 days	12 months
Veraldi S [5]	F/53	N	Leg	Ulcer	Potassium permanganate packs Topical gentamicin Silver sulfadiazine	Ciprofloxacin 750 mg PO qd + 0.05% sodium hypochlorite 2 packs qd for two weeks	10 months
Veraldi S [5]	M/75	A scratch from a bush	Leg	Ulcer	Simvastatin and allopurinol.	Ceftriaxone 2 g IV qd + sodium hypochlorite qd for 10 days.	8 months
Drago F [15]	M/79	N	Scrotum	Ulcer	N	Ciprofloxacin 500 mg PO tid for 5 days Ceftazidime 1 g IM tid for 7 days.	2 months
García FR [16]	N/10	N	Arm	Erythematous plaque	Commonly used antiseptics and topical antibiotics (bacitracin, fusidic acid)	Ciprofloxacin 500 mg q12h for 15 days	10 months
Seo J [8]	M/33	Laceration caused by a box cutter	Hand	Cellulitis	N	N	N
Seo J [8]	F/33	Augmentation rhinoplasty (unclear material)	Nose	Papular erosions	N	N	N

*Note:* bid, bis in die; F, female; IV, intravenous injection; M, male; N, not found; PO, per os; qd, quaque die; tid, ter in die.

## 5 Discussion

*Serratia. marcescens* is a well‐established nosocomial and opportunistic pathogen known for causing a diverse array of infections, including urinary tract, respiratory tract, and wound infections among hospitalized and immunocompromised patients [17]. The occurrence of cutaneous infections attributable to *S. marcescens* is extremely rare, especially in immunocompetent patients [4, 5]. Multiple factors may contribute to the refractory inflamed nodule. First of all, despite the low incidence rate in the aforementioned population, *S. marcescens* still exhibits a typical pattern in skin abscess formation similar to other anaerobic bacteria. These infections typically arise following minor trauma at sites prone to cavity formation. Findings from Seo et al. [8] and Park and Seo [10] so support this notion. In this particular case, filler injection can create a tunnel allowing entry of *S. marcescens*, and can also create a cavity which is lacks oxygen. Secondly, HA can support the growth of bacterial biofilm, which can become a shield against antibiotics [1, 18]. Thirdly, *S. marcescens* is notorious for its ability to acquire resistance to multiple antibiotics through various mechanisms [17, 19, 20]. Lastly, delayed use of proper antibiotics is another important reason for recurrence among all the cases we reviewed. In this case, once *S. marcescens* colonizes in the injection area, it will be difficult to cure.

Diagnosis of bacterial infection is usually composed of risk factors, clinical signs/symptoms, and pathogen examination. The clinical manifestations of skin infection caused by *S. marcescens* include nodules, cellulitis, dermal abscesses, and ulcers [6, 8, 12]. In this case, the patient presented swelling, hardening, reddening, tenderness, and pus discharge at the injection site. But similar conditions can also be seen in other pathogens such as *Staphylococcus aureus*, *Streptococcus pyogenes*, *Mycobacteria*, and *Escherichia coli* [21]. Due to the lack of specificity of signs and symptoms, it is difficult to diagnose without the help of pathogen examination when suspecting bacterial infection after filler injection. Besides, for patients who underwent long‐term antibiotic therapy before clinic, co‐infection should also be considered. Another differential diagnosis is a hypersensitive reaction, which can also present as inflammatory nodules at the injection site. However, given the long time span (18 months after the procedure) and positive pathogen examination in the late phase, a hypersensitive reaction is unlikely in this case [1, 22].

Before the discussion of antibiotics, it should be clear that the challenge of late infection caused by *S. marcescens* is multi‐drug‐resistant. Through the production of AmpC β‐lactamases, *S. marcescens* gets intrinsic resistance to penicillin and first‐/second‐generation cephalosporins [19, 20]. Moreover, a study found that *S. marcescens* has resistance genes to almost all kinds of antibiotics [17], which means that although *S. marcescens* is sensitive to many kinds of antibiotics such as third‐ and fourth‐generation cephalosporin, aminoglycosides, quinolones, and TMP‐SMX [6, 9, 10, 11, 12, 13, 14, 16], this pathogen has the potential to become resistant to more and more drugs in the future.

Third‐generation cephalosporin should be cautiously used. Because it may induce the generation of AmpC β‐lactamases, which can hydrolyze more β‐lactam antibiotics such as piperacillin, third‐generation cephalosporin, and aztreonam without being inhibited by β‐lactamase inhibitor [23]. This helps the bacteria escape from the pharmacological effect. Besides the process mentioned above, the majority of the reports we reviewed and our experience from this case also prove this statement. Although the usage of third‐generation cephalosporin is usually shown to be susceptible in most of the culture results, it is only partially successful in treating this kind of infection. By contrast, aminoglycosides, quinolone, or TMP‐SMX for at least 2 weeks usually achieves complete remission, which should be considered for the first‐line treatment [5, 6, 7, 8, 9, 10, 11, 12, 13, 14, 15, 16]. However, it should be noted that quinolones and carbapenems may cause selective pressure for multiresistant organisms, and aminoglycosides have potential ototoxicity and nephrotoxicity [24].

For patients with inflammation nodules at the injection site, evaluation of the lesion is the first move in the clinic. For erythematous nodules or tender lumps without fluctuation, hyaluronidase should not be used because of the risk of infection diffusion [25]. Once a fluctuant abscess is suspected, hyaluronidase can be administered to improve the clinical outcome by destroying the bacteria biofilm [26]. Also, followed by thorough debridement and drainage, pathogen examination and drug‐sensitive tests should be done. Despite some patients denying the possibility of immunocompromise, skin tests with tuberculin, radiographic chest studies, atypical mycobacterial cultures, serology testing for human immunodeficiency virus (HIV), hepatitis B virus, hepatitis C virus, and syphilis [9] should also be done to rule out this possibility. Once *S. marcescens* infection is diagnosed, sensitive antibiotics should be administered. Sensitive aminoglycosides, quinolone (especially moxifloxacin), or TMP‐SMX [1] should be considered before third‐generation cephalosporin. This review shows that both single and combination of antibiotics can result in a complete resolution of the lesion. Although we used combination therapy in this particular case considering the possibility of co‐infection [8] and biofilm formation [1], we are unable to make a more informed comment regarding this phenomenon, and it requires further study.

## 6 Conclusion

With the help of pathogen examination and drug‐sensitive tests, sensitive aminoglycosides, quinolone (especially moxifloxacin), or TMP‐SMX for at least 2 weeks can be considered as the first‐line treatment of late subcutaneous infection caused by *S. marcescens* following hyaluronic acid injection.

## Author Contributions

All authors contributed to the study conception and design.

## Conflict of Interest

The authors declare no conflicts of interest.

## Ethics Statement

The authors have nothing to report.

## Data Availability

The data that support the findings of this study are available from the corresponding author upon reasonable request.
